# Effects of Expanded Hemodialysis with Medium Cut-Off Membranes on Maintenance Hemodialysis Patients: A Review

**DOI:** 10.3390/membranes12030253

**Published:** 2022-02-23

**Authors:** Zhuyun Zhang, Tinghang Yang, Yupei Li, Jiameng Li, Qinbo Yang, Liya Wang, Luojia Jiang, Baihai Su

**Affiliations:** 1 Department of Nephrology, West China Hospital, Sichuan University, Chengdu 610041, China; 2020224020032@stu.scu.edu.cn (Z.Z.); 2017151621156@stu.scu.edu.cn (T.Y.); 2019326200014@stu.scu.edu.cn (Y.L.); 2019324020034@stu.scu.edu.cn (J.L.); 2019224025258@stu.scu.edu.cn (Q.Y.); 2021324020024@stu.scu.edu.cn (L.W.); jiangluojia@163.com (L.J.); 2 Med-X Center for Materials, Sichuan University, Chengdu 610041, China; 3 Med+ Biomaterial Institute of West China Hospital, Sichuan University, Chengdu 610041, China; 4 The First People’s Hospital of Shuangliu District, Chengdu 610200, China

**Keywords:** expanded hemodialysis, medium cut-off membrane, artificial kidney, internal filtration–backfiltration mechanism, middle molecules

## Abstract

Kidney failure is associated with high morbidity and mortality. Hemodialysis, the most prevalent modality of renal replacement therapy, uses the principle of semipermeable membranes to remove solutes and water in the plasma of patients with kidney failure. With the evolution of hemodialysis technology over the last half century, the clearance of small water-soluble molecules in such patients is adequate. However, middle molecules uremic toxins are still retained in the plasma and cause cardiovascular events, anemia, and malnutrition, which significantly contribute to poor quality of life and high mortality in maintenance hemodialysis patients. A new class of membrane, defined as a medium cut-off (MCO) membrane, has emerged in recent years. Expanded hemodialysis with MCO membranes is now recognized as the artificial kidney model closest to natural kidney physiology. This review summarizes the unique morphological characteristics and internal filtration–backfiltration mechanism of MCO membranes, and describes their effects on removing uremic toxins, alleviating inflammation and cardiovascular risk, and improving quality of life in maintenance hemodialysis patients.

## 1. Introduction

Kidney failure, also known as end-stage renal disease (ESRD), was recently defined by the Kidney Disease: Improving Global Outcomes Consensus Conference in terms of an estimated glomerular filtration rate below 15 mL/min/1.73 m^2^ or treatment with dialysis [1]. In 2017, the global prevalence of chronic kidney disease was estimated as 9.1%, with ESRD accounting for 0.07%, or approximately 5.3 million patients [2]. Beyond high morbidity, kidney failure is also associated with high mortality, resulting in 1.2 million deaths annually [2]. Currently, the number of patients receiving renal replacement therapy, either dialysis or renal transplantation, is more than 2.5 million [3], a number that continues to grow rapidly because of increased availability of dialysis, population ageing, and increased prevalence of hypertension and diabetes mellitus [4]. Hemodialysis, as the most prevalent modality of renal replacement therapy, uses the principle of semipermeable membranes to remove solutes and water in the plasma through different mass separation mechanisms, such as diffusion, convection, and ultrafiltration (UF) [5,6].

Along with the decrease in the estimated glomerular filtration rate, a number of uremic toxins are unable to be eliminated by the kidneys and still retained in the plasma. According to the molecular weight of these uremic toxins, they are divided into six classes, including small water-soluble molecules (<500 Da), protein-bound uremic toxins (PBUTs; mostly <500 Da), small-middle molecules (0.5–15 kDa), medium-middle molecules (15–25 kDa), large-middle molecules (25–58 kDa), and large molecules (>58 kDa) [7], as shown in Table 1. It has been well established that the accumulation of such toxins in the plasma is associated with cardiovascular risk [8], chronic kidney disease-mineral and bone disorders [9], neurologic manifestations [10], and inflammation [11], which significantly contribute to the poor quality of life and high mortality in maintenance hemodialysis patients.

Low-flux hemodialysis, as the traditional mode of dialysis in the past decades, provides efficient clearance of small water-soluble molecules, such as creatinine and urea [12], but negligible removal of middle molecules and protein-bound uremic toxins [13,14]. Compared with low-flux hemodialysis membranes, high-flux membranes have a higher UF coefficient, resulting from increased hydraulic permeability. As a result, high-flux hemodialysis with such membranes has a molecular weight cut-off (MWCO) of 25 kDa and has gradually become the most common mode of hemodialysis treatment worldwide [15]. In hemodiafiltration (HDF) mode, which combines convection and diffusion, molecules up to 30 kDa are efficiently removed [16]. The European Dialysis Outcomes and Practice Pattern Study confirmed that high-efficiency hemodiafiltration was associated with a 35% mortality risk reduction compared with low-flux hemodialysis [17,18]. However, hemodiafiltration therapy requires specialized equipment, well-functioning vascular access, remarkably high blood flow, and large volumes of ultrapure dialysate and sterile substitution fluid, thus is not suitable for all patients [19,20].

Further elimination of larger medium toxins in plasma is crucial to reduce mortality and improve prognosis. High cut-off (HCO) membranes [21,22,23] have a cut-off for protein permeability close to that of glomerular basement membrane, making it possible to clear large-middle molecules up to a molecular weight (MW) of 60 kDa [24] and PBUTs in blood. Therefore, HCO membranes have been applied as adjuvant treatment for patients with acute kidney injury secondary to multiple myeloma [25,26] and severe sepsis [27]. However, some studies found that the use of HCO membranes was associated with a large amount of albumin loss and even clinical hypoalbuminemia, which limited their routine application in chronic hemodialysis [28].

In this context, recent advances in the membrane manufacturing industry have led to the development of a novel class of dialysis membrane with MWCO close to MW of albumin and very high retention onset, previously called medium cut-off (MCO) and now defined as high retention onset (HRO) membrane, which could better reduce the circulating levels of middle molecules, while allowing albumin to remain in the plasma [13,29,30]. MCO membranes are structured by polyarylethersulfone/polyvinylpyrrolidone (PAES/PVP) and have a mean pore radius of 5 nm, between high-flux and HCO membranes [20]. Over the last decade, hemodialysis with such membranes in clinical practice has made it possible to perform a new therapy, called expanded hemodialysis (HDx), to deliver expanded removal of middle and large molecular solutes that are typically retained by current dialysis therapy and to improve outcomes for maintenance hemodialysis patients [30,31]. As diffusion and convection are conveniently combined inside a hollow fiber dialyzer equipped with MCO membranes during expanded hemodialysis therapy, the use of replacement solution is no longer required. Although the concept and prior clinical practice of expanded hemodialysis have been summarized in previous reviews, those were based on limited clinical experience and feedback, and lacked substantial evidence of clinical application [30,31]. As more clinical results become available over the years, the effects of expanded hemodialysis on patient-centered outcomes becomes clearer. Herein, we set out to systemically discuss the morphological characteristics and internal filtration–backfiltration mechanism of MCO membranes and describe their effects on removing uremic toxins, alleviating inflammation as well as cardiovascular risk, and improving quality of life in maintenance hemodialysis patients.

## 2. Unique Characteristics of Medium Cut-Off Membranes

### 2.1. Medium-Size Pore Radius and Tight Distribution of Pores

The process of manufacturing dialysis membranes significantly affects their morphological characteristics, such as mean pore size, pore size distribution, surface porosity, and pore tortuosity, which further influence the molecular-weight removal spectrum and membrane clearance [32]. Homogeneous mixtures of synthetic polymers and bore liquid, high-precision spinning technology, high-speed online drying process, high-precision precipitation conditions, and temperature control at each step deliver MCO membranes that have a permeability spectrum and selectivity and resemble the natural kidney more than the current high-flux membranes [32].

**Figure 1:**
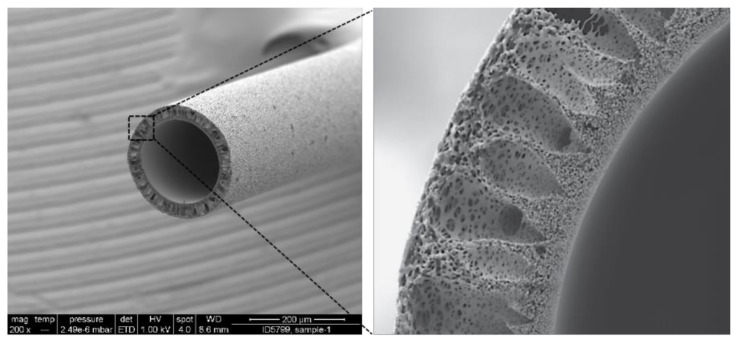
Scanning electron micrographs of fiber (**left**) and fiber wall (**right**). Pictures are from Baxter International, Inc., Deerfield, IL, USA.

Figure 1 shows the asymmetric three-layer structure of MCO membranes, which have an effective pore radius of 3.0–3.5 nm after contact with blood, allowing for the removal of an expanded range of uremic toxins (up to 45 kDa) [33]. Although MCO membranes have larger pore sizes, they effectively retain pyrogens, including endotoxins and other bacterial contaminants, at a similar level to other classic, less open, high-flux membranes [20,34]. Table 2 further shows the parameter characteristics of different dialyzers.

**Figure 2:**
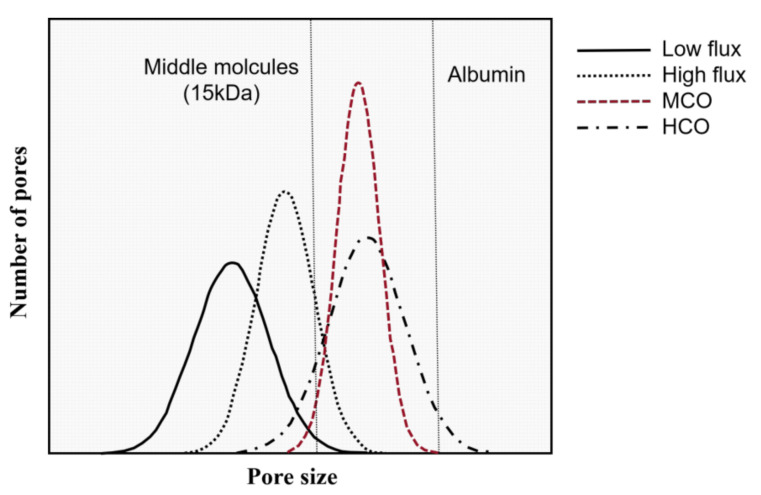
Schematic of pore size distribution in different types of membranes. As MCO membranes have been developed to increase the size of uremic-toxin molecules without albumin leakage, the distribution of pores has been tightened. Modified from Dr. Martin Wolley [35].

In addition, MCO membranes have a tighter pore distribution compared to high-flux and HCO membranes (Figure 2) [32,35]. The pores within these membranes are nonuniform, with a bell-shaped size distribution from small to large. To increase the size of molecules removed by MCO membranes without excessive albumin loss, the sizes of the pores are increased by moving their distribution to the right, but the largest pores are still smaller than albumin. Further, the distribution of pores within the dialysis membranes fundamentally changes to a tighter distribution to enable them to be more uniform than HCO membranes. This tight pore distribution enhances the permeability and selectivity of MCO membranes.

### 2.2. Steep Sieving Curve

The permeability characteristics in terms of sieving capacity were used to evaluate a dialysis membrane [36]. The sieving curve shows a progressive reduction in sieving value as the solute MW increases, until 90% of the solute is retained in the filtration process (where sieving = 0.1), and the corresponding MW of the solute defines the cut-off value of the membrane (MWCO). On the other side of the sieving curve, the MW at which only 10% of the solute is retained (where sieving = 0.9) defines the retention onset of the membrane (MWRO) [31,36].

**Figure 3:**
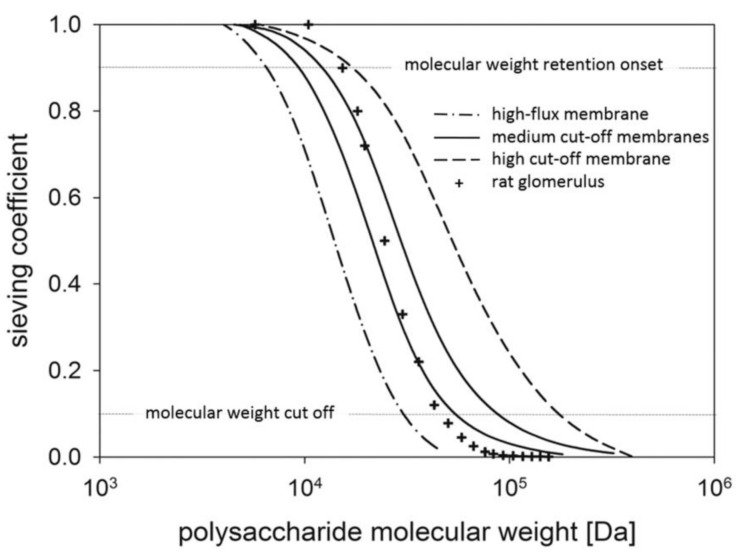
Sieve curves of different dialysis membranes and rat glomerulus. Sieving profile of high-flux, medium cut-off, and high cut-off dialysis membranes was determined by dextran filtration (as reported by Boschetti-de-Fierro et al. [13]). Data for glomerular membrane (as reported by Axelsson et al. [37]) were added for comparison (rat specimen, Ficoll filtration, measured in vivo). Picture is from Markus Storr [38].

Storr compared the sieve curves of different dialysis membranes [13] and the rat glomerulus [37] that were obtained by macromolecular polysaccharides with a broad molecular weight distribution (Figure 3) [38]. The sieving curve for MCO membranes is located at molecular weights between those of the conventional high-flux and HCO membranes and is similar to the Ficoll sieve curve of glomerular membrane, demonstrating that the permeability of MCO membranes is closest to that of natural kidney [38,39]. Furthermore, the tight pore distribution in MCO membranes leads to a steep sieving curve, characterized by high MWRO and MWCO values close to but lower than that of albumin. Thus, the novel MCO membranes were recently redefined as HRO membranes [31]. The unique sieving curve characteristic of MCO membranes may result in an improved removal of uremic toxins while limiting the loss of albumin.

### 2.3. Internal Filtration–Backfiltration Mechanism (IF-BF)

During the manufacturing process, MCO membranes require the hollow fiber diameter to be reduced from the standard 200 μm to 180 μm [30], which increases the wall shear rate and blood flow velocity [40], to avoid protein stagnation at the blood membrane interface and improve solute transport [41]. MCO membranes are thus characterized by higher permeability than classic high-flux membranes due to a remarkable amount of internal filtration (IF) in the proximal part [42], resulting from an increased end-to-end pressure drop. Therefore, the convective transport of MCO membranes increases by a large margin along the length of the fibers, which makes it possible to remove large molecules with low diffusion coefficients. Exogenous substitution fluid and complex set up are not required because there is an adequate amount of backfiltration (BF) [43] in the distal part of the hemodialyzer, almost covering the convective volumes [44,45]. Lorenzin [43] recorded dynamic imaging after injecting a non-diffusible marker molecule (albumin macro-aggregates labeled with ^99^Tc metastable) and successfully demonstrated the existence of an IF-BF mechanism even though it was not visible [41]. In vitro, the amount of filtration and backfiltration inside the Theranova 400 dialyzer was found to be more than 30 mL/min at the blood flow of 300 mL/min and at zero net filtration. It could reach values around 50 mL/min when there were higher blood flow, larger surface area, and higher net filtration rates (15–20 mL/min). Internal filtration between 30 and 50 mL/min combined with the sieving characteristics of MCO membranes allows for convective clearance values of medium-large molecules equal or even superior to those achieved in high-volume online HDF, without the need for fluid replacement and very high filtration fractions inside the hemodialyzer [30,36,41].

**Figure 4:**
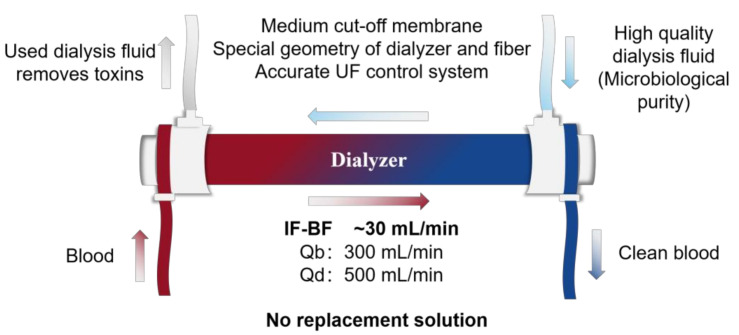
Requirements to perform expanded hemodialysis and related operational parameters (modified from Claudio Ronco) [31].

In the past, the only way to increase convective clearance was to increase the ultrafiltration rate, because the sieving of the selected molecule is low. The online HDF has made high convection rates possible thanks to the combined pre- and post-dilution configuration, but it requires complex hardware to ensure the purity of the substitution fluid. The emergence of expanded hemodialysis is currently being regarded as a breakthrough in terms of efficiency and simplification. The unique sieving properties and IF-BF mechanism allow MCO membranes to offer expanded clearance compared with high-flux membranes used in HD mode and equivalent clearance to high-flux membranes in high-volume HDF mode for uremic solutes in the 15,000 to 45,000 Da size range. As a result, HDx covers a removal spectrum that extends the current capabilities of the best therapy available. First, due to the increased sieving capacity and IF-BF mechanism, the ultrafiltration rate could be lower and the amount of required dialysis fluid could be retrenched to achieve the same solute clearance with HDF therapy. Second, there is no need for complicated equipment for online production of substitution fluid or well-functioning vascular access for high blood flows, except the ultrafiltration control system of regular hemodialysis machines, which provides the exact amount of net filtration required for the scheduled weight loss of dialysis patients. Blood flow ≥300 mL/min and dialysate flow ≥500 mL/min are sufficient to achieve optimal clearance in the system [31,36], as shown in Figure 4. The availability and applicability of MCO membranes in clinical practice may allow an update in the selection of dialysis techniques and raise the standard of treatment for all chronic hemodialysis patients.

## 3. The Effect of Expanded Hemodialysis on Uremic Toxins Removal

Recently, a growing number of studies have been published with the main aim of evaluating the efficiency of uremic toxin removal by expanded hemodialysis with MCO membranes. This section mainly summarizes the efficiency of this hemodialysis modality compared with high-flux hemodialysis and hemodiafiltration.

### 3.1. Dialysis Adequacy

Single-pool Kt/V_urea_ (spKt/V) is defined as urea clearance multiplied by duration of treatment and normalized for urea distribution volume, and is widely used to measure the adequacy of dialysis in clinical practice [46]. Current KDIGO guidelines recommend a target spKt/V of 1.20–1.40 per thrice-weekly dialysis session [47]. Among hemodialysis patients, a value of spKt/V less than 1.2 is associated with higher all-cause mortality [48,49]. Béguin established a predictive model of Kt in the upper quartile to estimate mortality, concluding that higher spKt/V was consistently associated with better survival [50]. Several small randomized controlled trials have consistently shown no difference in dialysis adequacy between MCO and high-flux membranes [51,52,53,54]. The latest prospective COREXH registry enrolling 992 hemodialysis patients further showed that HDx therapy significantly increased spKt/V from 1.62 at baseline to 1.70 during one year of follow-up [55].

### 3.2. Removal of β2 Microglobulin (β2-M)

β2-M has a molecular weight of around 11,800 Da and is well known as both a potential standard representative marker for middle molecule accumulation and a molecule with direct pathological consequences in the case of dialysis-associated amyloidosis [56,57]. Most studies showed that HDx therapy with MCO membranes led to a higher reduction ratio of β2-M compared with high-flux hemodialysis [52,53,54,58,59]. Many factors could influence the reduction rate of β2-M, such as blood flow rate, which was lower (about 250 mL/min) in Lim’s study [51], while it was targeted to 300 mL/min in other studies. In addition, the pre-dialysis level of β2-M decreased slightly and was sustained after three-month HDx in two crossover trials [53,54]. Nevertheless, in a prospective observational study, the serum level of β2-M rebounded even higher than that at baseline after treatment with HDx for one year [58]. The same phenomenon has been reported in hemodiafiltration [60]. Ward hypothesized that resistance to intercompartmental mass transfer explains this observation and limits β2-M removal [60]. In conclusion, whether medium cut-off dialyzers could lead to a sufficient long-term decrease of β2-M serum concentrations is an area of concern.

### 3.3. Removal of Free Light Chains (FLCs) and Other Middle Molecules

Polyclonal serum free light chains (FLCs), which are produced by cells of B-cell lineage and metabolized by the kidney, have a molecular weight of 22.5 and 45 kDa for kappa FLC (κ-FLC) and lambda FLC (λ-FLC), respectively [33]. It is now widely accepted that serum levels of FLCs are independently associated with mortality in patients with ESRD [61]. Consequently, FLCs have recently been proposed as a panel of biomarkers representing medium-middle and large-middle molecules that cannot be significantly removed by hemodialysis [7].

Recently, a multicenter, randomized controlled trial enrolling 172 patients on maintenance hemodialysis in the United States showed that the reduction ratio for the removal of both κ-FLC and λ-FLC was significantly higher in the HDx group with Theranova 400 dialyzers compared with conventional high-flux dialysis with Elisio-17H dialyzers after 24 weeks [52]. Furthermore, the Theranova 400 dialyzers demonstrated superior removal of other middle to large molecules such as complement factor D, TNFα, and β2-M. A sustained reduction in mean serum FLCs was also shown during the HDx phase, demonstrating that the effects of higher reduction of FLC were sustained until the following dialysis sessions. Likewise, the THE SHE (Theranova in Sisli Hamidiye Etfal) study, the REMOVAL-HD (tRial Evaluating Mid cut-Off Value membrane clearance of Albumin and Light chains in HemoDialysis patients) study, and a randomized clinical trial by Belmouaz, et al. with smaller sample size consistently demonstrated that circulating levels of κ-FLC and λ-FLC following hemodialysis were more pronounced when using MCO membranes as compared to high-flux membranes [53,54,62].

It is noteworthy that expanded hemodialysis with MCO membranes is also significantly associated with a higher myoglobin (17 kDa) reduction ratio than hemodialysis with high-flux membranes [53,54]. In summary, these short-term studies collectively show greater removal of larger middle molecules following the use of the MCO dialyzers than conventional high-flux dialyzers.

### 3.4. Removal of Protein-Bound Uremic Toxins (PBUTs)

PBUTs, including homocysteine, indoxyl sulfate (IS) and p-cresulfate (p-CS), are a group of molecules retained in dialysis patients. They are difficult for current dialyzers to remove due to albumin binding, in spite of light molecule weight (mostly <500 Da) [63]. Total plasma homocysteine levels in patients with ESRD are as much as three to four times greater than those in the general population, and elevated levels of total PBUTs are significantly linked to a range of pathological effects, including endothelial damage and cardiovascular disease [64,65]. A recent expert conference recommended that clearance of protein-bound solutes is best estimated by analyses of indoxyl sulfate and paracresyl sulfate although the elimination of these solutes might be affected by the residual kidney function of dialysis patients [7].

As discussed above, MCO membranes have a larger pore radius than conventional high-flux membranes, making it possible for them to remove more PBUTs by potential albumin leaking. However, recent clinical studies evaluating changes in protein-bound solutes following HDx have yielded conflicting results. An exploratory sub-study of the REMOVAL-HD trial, enrolling 89 participants, found no significant changes in total or free levels of IS or p-CS after 12 or 24 weeks of MCO membrane use compared to baseline, as no significant albumin loss was observed in this study [66]. In contrast, data from another randomized clinical trial by Belmouaz and his colleagues showed that expanded hemodialysis with MCO membranes was associated with a higher mean homocysteine reduction ratio compared with high-flux dialysis during a 3-month follow-up period [54]. Final answers will come from future studies, particularly large-scale randomized controlled clinical trials.

In conclusion, expanded hemodialysis with MCO membranes showed a greater capacity to remove middle-molecule toxins than high-flux dialysis. However, removal of these middle molecules is merely indicative of surrogate markers of potential clinical benefits of MCO dialyzers in ESRD patients and needs to be robustly evaluated in long-term randomized controlled trials with patient-centered outcomes.

In addition, compared to online HDF, recent small, short-term crossover clinical studies and a one-year retrospective study consistently suggested that expanded hemodialysis therapy was not inferior to online HDF in removing small and larger middle molecules, and thus it could be an alternative for patients who have no access to conventional hemodiafiltration therapy [16,33,67,68,69,70]. More importantly, long-term patient-centered outcomes of expanded hemodialysis compared with high-flux dialysis and hemodiafiltration should also be examined in future clinical trials.

## 4. The Effect of Expanded Hemodialysis on Inflammation and Cardiovascular Risk

### 4.1. Effect of Expended Hemodialysis on Inflammation and Oxidative Stress

A persistent microinflammation status and oxidative stress characterized by high plasma levels of inflammatory biomarkers with a molecular weight in the range of 15–50 kDa [71] (namely, C-reactive protein (CRP), interleukin 1β (IL-1β), interleukin 6 (IL-6) and tumor necrosis factor-α (TNF-α) [72,73]) and disturbances in pro-oxidant and anti-oxidant balance [74] are now considered hallmark features of kidney failure [75], which is significantly associated with malnutrition, cardiovascular events, and enhanced all-cause mortality in hemodialysis patients [76]. Increased reactive oxygen species (ROS) could lead to oxidation of lipids, proteins, and DNA [77].

Data from patients with sepsis-associated acute kidney injury suggested that the unique IF-BF mechanism of MCO membranes might play an important role in removing inflammatory cytokines [78]. Recently, a growing number of clinical studies have investigated the efficiency of expanded hemodialysis with the MCO membrane in the removal of inflammatory cytokines compared with conventional hemodialysis, as shown in Table 3. In 2017, a randomized crossover clinical trial designed by Zickler et al. found that the HDx significantly reduced the expression of proinflammatory cytokine (TNF-α and IL-6) mRNA in peripheral leukocytes to a significantly greater extent than high-flux dialyzers, illustrating that MCO membranes might reduce cytokines through affecting gene transcripts [79]. However, this difference was lost when the study was extended to 12 weeks, while there were also insignificant reductions in the plasma concentrations of these cytokines with MCO membranes. Two randomized controlled trials demonstrated that HDx therapy with MCO membranes was associated with an increased reduction ratio of TNF-α and a lower serum level compared with the high-flux group, although it was not the primary end point of their studies [52,80]. Furthermore, a crossover prospective study [54] found that HD treatment decreased oxidized low-density lipoprotein and superoxide dismutase activity levels, but concentrations of IL-1β, IL-6, and TNF-α in plasma did not change. Cozzolino and Sevinc observed similar negative results with regard to cytokines [53,81].

A recent prospective cohort study by Hasan, et al. enrolling 42 patients did not find any significant difference in C-reactive protein and oxidative stress levels among low-flux, medium cut-off, and high-flux membranes in the whole study group. However, in the subgroup analysis of patients who had high CRP levels at baseline, HDx therapy with MCO membranes could lead to a significant decrease in CRP levels when compared to low-flux and high-flux membranes (2.8 vs. 13.7 and 6.1 mg/L, respectively, *p* = 0.05) [82].

Altogether, expanded hemodialysis with MCO membranes might have good potential to promote inflammatory status in maintenance hemodialysis patients, although the results are inconsistent. Future randomized controlled trials with large populations are crucially required to determine the effect of MCO membrane on ameliorating inflammation and oxidative stress in patients on chronic hemodialysis.

### 4.2. Effect of Expanded Hemodialysis on Cardiovascular Parameters

Cardiovascular disease is the leading cause of morbidity and mortality in patients with ESRD [83,84,85]. In these patients, in addition to traditional cardiovascular risk factors (including hypertension, diabetes, dyslipidemia and aging), some nontraditional kidney failure-related factors, such as the presence of uremic toxins, electrolyte and fluid imbalance, endothelial dysfunction and vascular calcification, anemia, increased oxidative stress, and chronic inflammation [86], significantly contribute to worsening cardiovascular outcomes [87,88,89]. In fact, several uremic toxins (including FGF23, IS, p-CS, homocysteine, IL-1β, and IL-6) significantly correlate with vascular calcification, coronary atherosclerosis, and left ventricular hypertrophy [90,91,92]. As expanded hemodialysis with MCO membranes contributes to greater removal of such molecules, a growing number of studies have been performed to determine whether the use of MCO membranes will decrease the cardiovascular risk of dialysis patients.

Willy incubated calcifying vascular smooth muscle cells with serum samples from chronic dialysis patients, and found that the degree of calcification and the concentration of calcification-associated proteins, such as matrix Gla protein (MGP), osteopontin (OPN) and GDF-15, were significantly reduced in the MCO group compared to the high-flux group [93]. Besides, the apoptosis rate of cells incubated with dialysate from the HRO group was 15% lower compared to high-flux incubated cells. It was concluded that HDx therapy could reduce the pro-calcific potential of serum from dialysis patients in vitro [94].

A prospective observational study with 12-month follow-up investigated the effect of MCO membranes on GDF15, sclerostin, and FGF23, which correlate with cardiovascular complications in patients with ESRD. MCO membranes had higher reduction ratios of these molecules than high-flux membranes, although hard endpoints, such as myocardial infarction and heart failure, were not used [95]. In addition, a recent randomized controlled trial by Lee, et al. enrolling 80 patients compared several cardiovascular parameters between patients undergoing HDx and online HDF [96]. The primary endpoints were changes in brachial–ankle pulse wave velocity (baPWV), echocardiographic parameters, and coronary artery calcium (CAC) scores over 1 year, and the secondary endpoints included blood cardiovascular biomarkers, mortality, and patient-reported outcomes. The results showed that HDx with MCO membranes was not inferior to online HDF in terms of baPWV and echocardiographic parameters. Most importantly, cardiovascular and all-cause mortality were similar between the two groups.

Remarkably, a distinct increasing trend in CAC scores was shown in the HDx group, so care should be taken when dialytic patients with a high CAC score or a score with an increasing trend have conventional hemodialysis or online HDF replaced with HDx [96].

## 5. Effect of Expended Hemodialysis on Quality of Life (QoL)

Patients receiving maintenance hemodialysis suffer from considerable physical symptoms, such as post-dialysis fatigue, itching, and cramping, which can adversely affect their quality of life [97]. The evaluation of quality of life (QoL) includes two domains, mental and physical health [98], and it is also widely accepted that QoL is an independent predictor of risks of ESRD and mortality [99].

Long-term HD patients may benefit from MCO dialyzers and have somewhat improved QoL, as MCO membranes tend to remove more large-middle molecules than conventional high-flux dialysis, although the association between large-middle molecule clearance and QoL remains unknown. The COREXH registry, a prospective multicenter observational cohort study in Colombia, enrolled 992 dialysis patients who switched from high-flux dialysis to HDx therapy. Changes in Kidney Disease Quality of Life 36-Item Short Form Survey (KDQoL-SF36) domains, Dialysis Symptom Index (DSI) scores, and restless leg syndrome (RLS) symptoms were recorded during the 12 months of follow-up. The results showed that three of five KDQOL-SF36 domains, symptoms, effects of kidney disease, and burden of kidney disease, improved compared with baseline and the proportion of patients diagnosed with RLS significantly decreased from 22.1 to 10% during 12 months of follow-up, suggesting that the expanded clearance of large-middle molecules provided by MCO membranes might be associated with improvements in patient QoL [100]. In a randomized prospective controlled open-label phase 4 trial, MCO dialyzers significantly improved patient-reported outcomes, particularly the physical components of QoL and uremic pruritus, compared to patients on high-flux dialyzers after 12 weeks of HDx therapy [51]. Similar findings were also obtained in the REIS study [101]. It is hypothesized that the improvement in patient QoL benefits from the more effective removal of large middle molecules provided by MCO membranes because the studies mentioned above also found expanded clearance in the MCO group [51,100]. The effect was modest but consistent across the full follow-up period.

In contrast, another multicenter randomized controlled trial by Weiner that assessed QoL in patients treated with HDx using KDQoL-36 and Europe QoL (EQ-5D-5 L) instruments over 24 weeks showed no significant differences between the HDx and high-flux groups [52]. Similarly, the REMOVAL-HD study in Australia and New Zealand found no significant improvement in QoL measured by the Edmonton Symptom Assessment System Revised (ESAS-r), RLS symptoms, and functional status over a 6-month period [62].

Altogether, whether expanded hemodialysis treatment with MCO membranes can improve the QoL of patients is not clear. In the future, studies with large samples, long-term follow-up, and better design are needed to verify whether better middle-molecule uremic toxins removal might influence QoL and ultimately life expectancy.

## 6. Health Economics

Patients with kidney failure pose a heavy healthcare burden worldwide because of the long-term use of renal replacement therapy and medications, such as erythropoietin-stimulating agents and antihypertensive drugs [102]. Expanded hemodialysis with MCO membranes may have the potential to alleviate the economic burden of ESRD by reducing medication usage and hospitalizations.

### 6.1. Medication Costs for Erythropoietin-Stimulating Agents

In a prospective crossover study including 40 patients randomly assigned to receive either 3 months of HDx followed by 3 months of high-flux dialysis or vice versa, the parameters of iron transport and metabolism, use of intravenous iron and erythropoietin-stimulating agents (ESAs) during HDx and high-flux periods and the erythropoietin resistance index (ERI) were recorded [54]. The proportions of iron and ESA usage were similar between the two groups, but the dosages were reduced, although the difference was not significant.

Surprisingly, in a multicenter observational cohort study [103,104] and a randomized controlled trial [80], patients in the HDx groups showed significant decreases in intravenous iron dose, ESA dose, ERI, and medication-related estimates of cost per patient year, and significant increases in levels of serum iron and transferrin saturation (TSAT) during the follow-up period compared with those in the high-flux groups. Multivariate linear regression analysis further confirmed that HDx therapy with MCO membranes was independently associated with decreased ERI [80]. The improved ESA resistance may be attributed to the improved removal of inflammatory cytokines and hepcidin [105,106].

### 6.2. Hospitalization Rates and Costs

A cohort study in Colombia, enrolling 81 hemodialysis patients who switched from high-flux to expanded hemodialysis for a year, found that the number of hospital days per patient-year significantly decreased from 5.94 to 4.41, while the hospitalization rate for cardiovascular causes and the 30-day readmission rate per patient-year were not significantly different between the HDx and high-flux groups, which might be attributed to the small sample size and potential for bias of the “before and after” study design [103]. Notably, estimates of average annual cost of hospitalization were nearly 24% lower with HDx [104]. Further work now needs to be done with muti-year studies using larger cohorts and control arms to confirm these preliminary results and explore other potential benefits.

In addition, expanded hemodialysis with MCO membranes does not require large volumes of sterile substitution fluid due to the unique IF-BF mechanism and could be applied in conventional dialyzers with an ultrafiltration-control system, without complex operations. The optimization of dialysis membranes and operations in expanded hemodialysis may lead to substantial cost savings.

## 7. Safety Concerns

### 7.1. Retention of Serum Albumin

Hypoalbuminemia is associated with nutritional status in ESRD patients along with the cardiovascular disease and the presence of inflammation [107]. The application of HCO membranes is limited because of albumin leakage. MCO membranes are characterized by a high MW cut-off value close to albumin, therefore, the albumin loss should be taken into consideration. Many researchers have recorded the changes in the level of serum albumin through the follow-up period.

A randomized controlled clinical trial conducted by Weiner in 21 centers in the United States recruited 172 participants and found maintenance of serum albumin levels following treatment with the Theranova 400 dialyzer, with superior removal of larger middle molecules compared with a similarly sized high-flux dialyzer [52]. Similar results were also found in other small preliminary clinical studies [62,68,108,109].

In contrast, some crossover trials observed reduced albumin levels to various degrees with HDx treatment [53,54,81], as shown in Table 4. Zickler reported that albumin levels dropped significantly from 3.7 to 3.53 g/dL after 4 weeks of HDx, but went back up during extended 8-week therapy [79]. In a large observational single-cohort study enrolling 638 patients who received HDx therapy, the maximum average change in mean serum albumin levels was 3.5% at the third month, while mean serum albumin levels remained within the normal range through 12 months [55].

It is noteworthy that the albumin loss that occurred with the MCO dialyzers was within the range observed in HDF treatment [110], which is less than transperitoneal albumin losses seen in peritoneal dialysis [111], and seemed to be limited compared to HCO therapy [112]. Moreover, no patients developed hypoalbuminemia or required additional albumin supplementation, suggesting that the serum albumin decline with MCO treatment is tolerable. It is possible that the quantity of albumin lost during expanded hemodialysis could be compensated rapidly by reflected higher hepatic synthesis.

On the other hand, a slight decrease in serum albumin might be beneficial for maintenance hemodialysis patients. Expanded hemodialysis might induce a moderate removal of PBUTs, oxidized albumin, and carbamylated albumin along with the serum albumin loss [113].

In expanded hemodialysis, the trend toward lower serum albumin levels may be an inevitable consequence of MCO membranes with the capacity to remove middle-molecule toxins close to the molecular size of albumin. It is also unknown whether a lower serum albumin level caused only by removal during hemodialysis leads to increased mortality.

### 7.2. Effects on Medication Clearance

Normally, drugs are removed through the glomerulus in healthy individuals, the process of which is variable in hemodialysis patients who have poor renal function. Compared with standard high-flux dialyzers, the removal of middle uremic toxins has improved with MCO membranes, but it is not known whether the increased pore size affects the retention of commonly used medications or coagulation factors in dialysis patients. Therefore, studies should pay close attention to the effect of MCO dialyzers on drug removal to ensure therapeutic levels and adjust medication dosages in time.

Using an in vitro model, the retention of erythropoietin, heparin, insulin, and several coagulation factors (factors II, VII, and X, protein C, and antithrombin III) with HDx compared with other types of dialysis was investigated [114]. The results showed that removing these drugs and molecules with HDx was comparable with high-flux and hemodiafiltration therapy, suggesting that it is not necessary to change the medication dosing or anticoagulation protocols for dialysis patients receiving HDx therapy with MCO membranes. However, more in vivo studies are needed to confirm this conclusion.

Allawati et al. further compared the clearance of vancomycin during hemodialysis with MCO membranes and high-flux membranes in a single-center, crossover clinical study [115]. Among the 210 study samples, vancomycin clearance was higher in the MCO group compared to high-flux the group, but it was not statistically significant. The median percentage of vancomycin removal at 120 min with MCO membranes was 39% (20.6–51.5%) compared to 34.1% (21.3–48.4%) with high-flux membranes. Therefore, the use of vancomycin during the last one to two hours of each HDx session is required to maintain therapeutic concentrations and minimize loss through the dialyzer.

### 7.3. Adverse Events

According to the experimental results that have been published thus far, no more adverse events or serious adverse events related to hemodialysis procedures after switching to HDx were reported, especially related to MCO membranes. It is possible that clinical studies with adverse events have not been published, so we expect more completed clinical studies to be published.

## 8. Conclusions and Prospects

In conclusion, MCO membranes have medium apertures, uniform distribution of pores, high MWRO and MWCO value close to but lower than that of albumin, a steep sieve curve close to the native kidney, and internal filtration–backfiltration mechanism. Because of these unique morphological characteristics, expanded hemodialysis with MCO membranes shows greater capacity to remove middle-molecule toxins than high-flux dialysis and is not inferior to online HDF, without loss of serum albumin. Along with removal of more middle molecules, expanded hemodialysis might alleviate the status of micro-inflammation and oxidative stress to some degree, partly improve quality of life and patient-reported outcomes, and have the potential to reduce cardiovascular risk and the economics burden by decreasing hospitalizations and medication usage. The application of expanded hemodialysis could allow clinicians to gain the benefits of HDF while using regular HD equipment, without requiring large amounts of high-quality fluid and a more complex setup. For the moment, expanded hemodialysis cannot completely replace high-flux or online HDF but can be a complement to hemodialysis.

However, the development and promotion of expanded hemodialysis still face many challenges. Despite the potential of expanded hemodialysis, further clinical trials need to take into account the complexity of dialysis patients to confirm its exact benefits. In addition, the removal of PBUTs and large-molecule uremic toxins is not solved with expanded hemodialysis. Therefore, the development and evolution of HRO membranes could improve the clearance of middle molecules more than 45 kDa in size and achieve the closest artificial kidney to natural kidney physiology. HRO membranes may play an important role in the advancement of expanded hemodialysis.

In this review, we focused on Baxter membranes, the Theranova 400 and Theranova 500 devices. At present, Fresenius Medical Care also produces membranes with similar functions. However, EMiC^®^2, produced by Fresenius, has been reported to treat acute diseases, such as acute renal failure due to sepsis or rhabdomyolysis by continuous renal replacement therapy, not maintenance hemodialysis. Moreover, there are fewer than 10 clinical studies on Fresenius membranes. Still, we expect great breakthroughs in Fresenius membranes and exciting clinical feedback on them.

Currently, many clinical studies on expanded hemodialysis therapy have been registered and are in the phase of recruiting patients. We expect that randomized controlled clinical trials with larger populations will validate the results we have obtained so far. In the future, we will have a new understanding of expanded hemodialysis therapy with MCO or HRO membranes.

## Figures and Tables

**Table 1:** Classification of uremic toxins and their representative biomarkers, respectively.

Uremic Toxin Class	Molecular Weight (kDa)	Representative Biomarkers
Small water-soluble molecules	<0.5	Urea (60 Da), creatinine (113 Da), uric acid (168 Da)
Small-middle molecules	0.5–15	PTH (9.5 kDa), β2-MG (11.8 kDa), cystatin C(13.3 kDa)
Medium-middle molecules	15–25	Myoglobin (17 kDa),TNF-α (17 kDa), sTNFR2 (17 kDa), IL-10 (18 kDa), FGF-2 (18 kDa), prolactin (22 kDa), κ-FLC (22.5 kDa), complement factor D (23.75 kDa), IL-18 (24 kDa), IL-6 (24.5 kDa)
Large-middle molecules	25–58	sTNFR1 (27 kDa), FGF-23 (32 kDa), VEGF (34.2 kDa), YKL-40 (40 kDa), λ-FLC (45 kDa)
Large molecules	>58	AOPP (>60 kDa), modified albumin (65 kDa)
Protein-bound uremic toxins	mostly < 0.5	Homocysteine, IS, pCS

PTH, parathyroid hormone; β2-MG, β2-microglobulin; TNF, tumor necrosis factor; sTNFR, soluble tumor necrosis factor receptor; IL, interleukin; FGF, fibroblast growth factor; κ-FLC, κ free light chains; VEGF, vascular endothelial growth factor; YKL-40, chitinase-3-like protein 1; λ-FLC, λ free light chains; AOPP, advanced oxidative protein products; IS, indoxyl sulfate; pCS, p-cresyl sulfate.

**Table 2:** Comparison of four dialyzer types in parameter characteristics. Information is from instructions of Baxter International, Inc., Deerfield, IL, USA.

Device	Membrane Type	Structural Characteristics				
		Pore Radius * (nm)	Fiber Inner Diameter (μm)	Fiber Wall Thickness (μm)	Effective Surface Area (m^2^)	UF-Coefficient ** (mL/h/mmHg)
Pollyflux 17L	Low-flux	3.1 ± 0.2	215	50	1.7	12.5
Revaclear 400	High-flux	3.9 ± 0.1	190	35	1.8	54
Theranova 400	Medium cut-off	5.0 ± 0.1	180	35	1.7	48
Theranova 500	Medium cut-off	5.0 ± 0.1	180	35	2.0	59
Theralite 2100	High cut-off	10.0 ± 2.0	215	50	2.1	52

* Pore radius: effective Stokes-Einstein radius, calculated from MWCO measured with polydisperse dextran. ** UF-coefficient: measured with bovine blood, hematocrit (Hct) 32%, and procalcitonin (Pct) 60 g/L, 37 °C.

**Table 3:** Effect of MCO membranes on micro-inflammatory status and oxidative stress.

Year	First Author	Patients (N)	Dialysis Treatment	Time	Study Design	Cytokines Significantly Removed by MCO Pre-Post Dialysis	Cytokines Significantly Removed by MCO at End of Study Period	Cytokines Removed by MCO Pre-Post Dialysis but No Significance	Reference
2017	Zickler	48	HD MCO vs. HF	12 weeks	4-week MCO 4-week HF pre-post dialysis 8-week extension	TNF-α mRNA IL-6 mRNA sTNFR1	TNF-α mRNA IL-6 mRNA sTNFR1	-	[79]
2019	Belmouaz	40	HD MCO vs. HF	6 months	3-month MCO 3-month HF pre-post dialysis	Homocysteine	Homocysteine	IL-1b, IL-6, TNF-a, Ox-LDL, 8-iso-Prostaglandin F2a, SOD activity	[54]
2019	Cozzolino	20	HD MCO vs. HF	6 months	3-month MCO 3-month HF pre-post dialysis	-	-	IL-1b, IL-6, TNF-α	[81]
2020	Lim	49	HD MCO vs. HF	12 weeks	12 weeks	TNF-α	TNF-α	-	[80]
2020	Sevinc	52	HD MCO vs. HF	6 months	3-month MCO 3-month HF pre-post dialysis	VEGF	VEGF	FGF-23, IFN-γ, IL-6, IL-10, IL-17A	[53]
2020	Weiner	172	HD MCO vs. HF	24 weeks	24 weeks	TNF-α	TNF-α	IL-6	[52]
2020	Yeter	42	HD MCO vs. HF vs. LF	6 months	6 months	-	-	TOS, TAS, PON-1, CRP	[82]

MCO, medium cut-off; HD, hemodialysis; HF, high-flux; LF, low-flux, TNF, tumor necrosis factor; RNA, ribonucleic acid; sTNFR, soluble tumor necrosis factor receptor; IL, interleukin; Ox-LDL, oxidized low-density lipoprotein; SOD, superoxide dismutase; VEGF, vascular endothelial growth factor; FGF, fibroblast growth factor; IFN, interferon; TOS, total oxidant status; TAS, total antioxidant status; PON-1, paraoxonase-1; CRP, C-reactive protein.

**Table 4:** Negative effect of MCO membranes on albumin removal. Researchers observed significantly decreased plasma albumin in these studies.

Year	First Author	Sample Size	Intervention	Time	Study Design	Pre-Dialysis Albumin Level (g/dL, Baseline vs. End)	Percentage Reduction	Reference
2017	Zickler	48	HD MCO vs. HF	12 weeks	4-week MCO 4-week HF pre-post dialysis 8-week extension	3.70 ± 0.36 3.53 ± 0.37	4.50%	[79]
2019	Belmouaz	40	HD MCO vs. HF	6 months	3-month MCO 3-month HF pre-post dialysis	3.71 ± 0.31 3.69 ± 0.43	-	[54]
2019	Cozzolino	20	HD MCO vs. HF	6 months	3-month MCO 3-month HF pre-post dialysis	3.8 (3.30–4.20) 3.6 (2.98–3.90)	5.20%	[81]
2020	Sevinc	52	HD MCO vs. HF	6 months	3-month MCO 3-month HF pre-post dialysis	3.88 (3.71–4.04) 3.62 (3.45–3.88)	6.70%	[53]
2020	Bunch	638	MCO	12 months	12 months	4.05 (4.04–4.07) 3.98 (3.96–4.00)	1.70%	[55]

## Data Availability

Not applicable.
